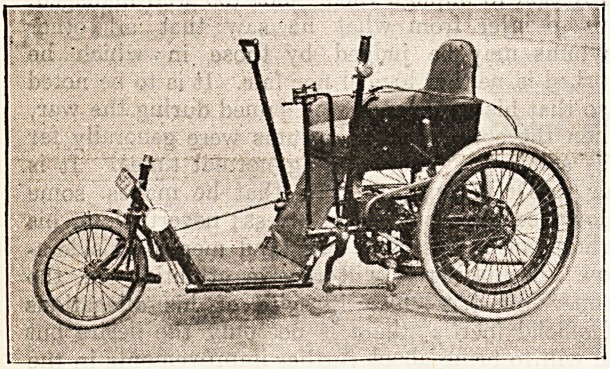# Institutional Needs

**Published:** 1921-08-13

**Authors:** 


### Institutional Needs.

### A New Motor and Hand-Propelled Tricycle.

This machine claims to provide advantages which are possessed by no other. It is primarily a standard model, hand-propelled tricycle, but is fitted with a petrol motor auxiliary unit, and this gives a speed of from two to fifteen miles per hour. The invalid or injured is able to climb hills of any gradient. The engine is easily started by two or three motions of the hand-levers, the hand-clutch is then released, the propelling-levers remain stationary, and the machine is under perfect control.

The machine is able to climb hills of any gradient, and one of its most useful features is that in the event of the motor not functioning for any reason, the rider is not left stranded, but is able to propel the machine home by means of the hand-levers with the greatest ease—a very important point in connection with tricycles specially designed for persons who are deprived of the use of the legs. It is manufactured by Carters, Ltd., the well-known invaids' furniture and appliance manufacturers, of 125/9 Great Portland Street, W. 1.

### Marking Ink.

There is no friend like an old one, particularly when the old one takes pains to retain friendship. This is the case with John Bond's "Crystal Palace" Marking Ink, which can always be depended on. Even the delicate attentions of the laundry do not affect it, and the more it is washed and boiled the more intense should its colour become. It has a reputation of over a century, and, like a certain famous gentleman, is still "going strong." It can be obtained in the heat or non-heat form and used either with pen, rubber stamp, or stencil. It is put up in 6d. and 1s. bottles, and in quarter, half, and one pint bottles. With the 1s. bottle is a linen stretcher and special marking-ink pen.

### Ultratan Catgut on Souttar Needles.

For some time, as is generally known in the hospital world, the ligature department of the London Hospital have been marketing, through Messrs. Allen & Hanburys, the various forms of suture material in surgical use, as the plant they have installed is capable of turning out a great deal more than is required for the work of the hospital alone. The latest development of this branch of institutional trading is the production of extra-durable catgut for intestinal work, ready threaded on intestinal needles, which are specially constructed so that in sewing with them the thread is pulled through single instead of double, as with all needles made with eyes in the usual way. We have already put to the test of practical experience the London Hospital catgut, with uniformly satisfactory results: with this new added "gadget," it should extend the popularity which it has already widely obtained.

## Figures and Tables

**Figure f1:**